# Dose response of a novel exogenous ketone supplement on physiological, perceptual and performance parameters

**DOI:** 10.1186/s12986-020-00497-1

**Published:** 2020-09-29

**Authors:** Philip J. Prins, Dominic P. D’Agostino, Christopher Q. Rogers, Dana L. Ault, Gary L. Welton, Dalton W. Jones, Samuel R. Henson, Tyler J. Rothfuss, Kylie G. Aiken, Jantzen L. Hose, Emilia L. England, Adam D. Atwell, Jeffrey D. Buxton, Andrew P. Koutnik

**Affiliations:** 1 Department of Exercise Science, Grove City College, 100 Campus Drive, Grove City, PA 16127 USA; 2 Department of Molecular Pharmacology and Physiology, University of South Florida, Tampa, FL USA; 3 Human Health, Resilience, and Performance, Institute of Human and Machine Cognition, Pensacola, FL USA; 4 Department of Psychology, Grove City College, Grove City, PA USA

**Keywords:** Ketone bodies, Ketogenic, Beta hydroxybutyrate, Medium chain triglycerides, Performance, Cognition

## Abstract

**Background:**

Interest into the health, disease, and performance impact of exogenous ketone bodies has rapidly expanded due to their multifaceted physiological and signaling properties but limiting our understanding is the isolated analyses of individual types and dose/dosing protocols.

**Methods:**

Thirteen recreational male distance runners (24.8 ± 9.6 years, 72.5 ± 8.3 kg, VO_2max_ 60.1 ± 5.4 ml/kg/min) participated in this randomized, double-blind, crossover design study. The first two sessions consisted of a 5-km running time trial familiarization and a VO_2max_ test. During subsequent trials, subjects were randomly assigned to one (KS1: 22.1 g) or two (KS2: 44.2 g) doses of beta-hydroxybutyrate (βHB) and medium chain triglycerides (MCTs) or flavor matched placebo (PLA). Blood *R*-βHB, glucose, and lactate concentrations were measured at baseline (0-min), post-supplement (30 and 60 min), post-exercise (+ 0 min, + 15 min). Time, heart rate (HR), rating of perceived exertion (RPE), affect, respiratory exchange ratio, oxygen consumption (VO_2_), carbon dioxide production, and ventilation were measured during exercise. Cognitive performance was evaluated prior to and post-exercise.

**Results:**

KS significantly increased *R*-βHB, with more potent and prolonged elevations in KS2, illustrating an administrative and dosing effect. *R*-βHB was significantly decreased in KS1 compared to KS2 illustrating a dosing and exercise interaction effect. Blood glucose elevated post-exercise but was unchanged across groups. Blood lactate significantly increased post-exercise but was augmented by KS administration. Gaseous exchange, respiration, HR, affect, RPE, and exercise performance was unaltered with KS administration. However, clear responders and none-responders were indicated. KS2 significantly augmented cognitive function in pre-exercise conditions, while exercise increased cognitive performance for KS1 and PLA to pre-exercise KS2 levels.

**Conclusion:**

Novel βHB + MCT formulation had a dosing effect on *R-*βHB and cognitive performance, an administrative response on blood lactate, while not influencing gaseous exchange, respiration, HR, affect, RPE, and exercise performance.

## Introduction

Ketones bodies are metabolic end products of lipid metabolism traditionally produced during fasting/starvation, severe caloric restriction, or low carbohydrate diets [1]. Endogenous ketone production is predominantly regulated via insulin and glucagon levels on adipose and hepatic tissue, respectively [2]. However, dietary restriction can be difficult for some to follow [1]. Consequently, exogenous ketone bodies have been developed to circumvent traditional barrier to elevating circulating ketone bodies [1, 3, 4]. Currently, available exogenous ketone bodies include medium chain triglycerides (MCT), beta-hydroxybutyrate (βHB)-salts and/or amino acids, and esters, all commercially available and generally recognized as safe (GRAS approved) by FDA. MCTs are six to twelve carbons in length and typically derived from natural food products [1, 5]. MCTs enter hepatic portal circulation and are rapidly metabolize to acetyl CoA resulting in subsequent hepatic ketogenesis and elevations in circulating βHB. βHB-salts or -amino acids are synthetically derived βHB molecules which are stabilized via chemical bonds electrolytes (primarily sodium) and/or amino acid. βHB-salts and/or -amino acid directly elevated circulating levels of βHB without hepatic metabolism. Esters have a synthetic 1,3 Butanediol backbone esterified to βHB as 1,3 Butandiol-βHB Monoester or AcAc as 1,3 Butanediol Acetoacetate Diester. Gastric esterases cleave βHB and/or AcAc from 1,3 butanediol backbone, directly elevating βHB and/or AcAc. The 1,3 butanediol backbone enters hepatic circulation and is subsequently catabolized via alcohol and aldehyde dehydrogenases into βHB. Consequently, all exogenous ketone bodies result in subsequent elevations in circulating ketone body elevations, but with diverse kinetics, tolerance, and application impact [1, 3, 6, 7].

Sport and/or athletic practitioners have been interested in exogenous ketone bodies dating back for almost a half-century when MCT’s their potential metabolic and performance impact in isolation or combination with other exogenous nutrients under the hypothesis that providing additional and diverse metabolite availability may augment sport related performance outcomes [5]. Mixed results and acute gastrointestinal barriers attenuated interest. However, in 2016, Cox et al. seminally described that 1,3 Butanediol-βHB Monoster administration regulated systemic and tissue specific metabolism, which results in physical performance enhancement in athletic cyclists [8]. Concurrently, ketone bodies were demonstrated to induce multifaceted effects across physiologic and cellular signaling which include metabolic, inflammatory, oxidative stress, epigenetic, immune, etc.… regulation [9]. Consequently, exogenous ketone bodies are now being explored for various health, disease, and performance applications [7, 10–12].

The physiologic, perceptual, and/or performance impact of exogenous ketone bodies’ have been mostly confined to the administration of individual types [5, 8, 13–23]. However, Kesl et al. and Ari et al. illustrated unique metabolic kinetics when co-administering diverse forms of exogenous ketones [3, 24]. Of interest were the findings that βHB-salts + MCTs augmented and prolonged elevations in circulating βHB compared to βHB-salts alone, illustrating unique metabolic impact [3]. Additionally, this unique mixture lowers the acute mineral and MCT load, potentially mitigating gastrointestinal impact [1]. Consequently, our group explored the metabolic and performance impact of βHB-salts and MCTs on 5-km running in well trained runners and found that this was well tolerated, altered systemic metabolism, and augmented and attenuated 8/10 and 2/10 5 k runner performance, respectively [25]. However, furthering limiting our knowledge on the impact of exogenous ketones bodies, is that almost all human trials has explored single dose/dosing protocols [5, 8, 13–23] limiting our understanding on the potential convergent and/or divergent dose–response across physiologic, metabolic, and performance parameters, with limited exception [6, 8]. Thus, we explored both the administration and dose response of βHB-salt and MCT on physiological, perceptual, as well as physical and cognitive performance parameters.

## Methods

### Study design

A randomized, double-blind, placebo-controlled, cross-over design was employed consisting of five separate laboratory visits. During their first visit to the laboratory each participant underwent a familiarization session during which participants were informed of all experimental procedures and familiarized with all performance measures to reduce the possibility of a learning effect. The familiarization trial was identical to experimental trials except that participants consumed no supplement prior to exercise. During the second visit, each participant’s maximal oxygen consumption (VO_2_max) was determined using a progressive multistage treadmill running protocol.

**Fig. 1:**
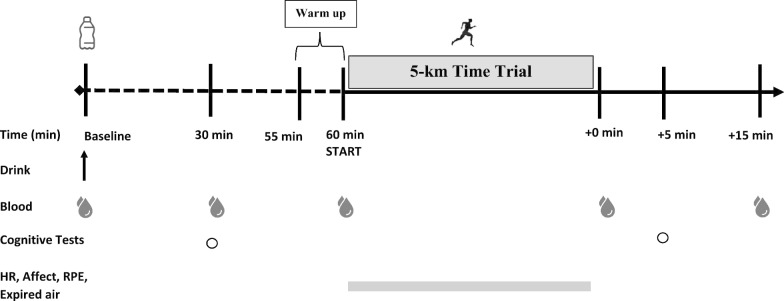
Study design. Subjects consumed beta hydroxybutyrate (βHB)-salts and medium-chain triglycerides (MCTs; KS1 and KS2) or flavor match control (PLA). Capillary metabolites were assessed pre-drink (baseline), 30 min post-drink (30 min), 60 min post-drink (60 min), post-run (+ 0 min), and 15 min post-5 k TT (+ 15 min). Cognitive tests were performed 30 min post-drink (30 min) and 5 min after 5 k TT (+ 5 min). Heart rate (HR), affect, rate of perceived exertion (RPE), and gas exchange were evaluated during the 5 k TT. Beta Hydroxybutyrate, βHB; Medium Chain Triglycerides, MCT; KS1, one dose βHB-salts and MCTs; KS2, two doses βHB-salts and MCTs; Flavor Matched Control, PLA; Heart Rate, HR; Rate of Perceived Exertion (RPE)

During subsequent visits, the three main experimental trials consisted of a 5-km running time trial (TT) with cognitive tests before (30 min) and after (+ 5 min), and were completed in a randomized (www.randomizer.org) counterbalanced sequence separated by 7 days. On experimental days, participants consumed either one (KS1: 22.1 g) or two (KS2: 44.2 g) servings of the ketone supplement (βHB + MCT) or a flavor matched placebo (PLA) drink 60 min prior to performing a 5-km running TT on a treadmill. Capillary glucose, lactate, and ketones were measured at baseline, 30 min post supplement ingestion (pre-cognitive test battery), 60 min post supplement ingestion, immediately following the TT (+ 0 min), and 15 min following the TT (immediately following the cognitive test battery). Other variables that were measured include (a) 5-km running time, (b) RPE (RPE-Overall; RPE-Chest; RPE-Legs), (c) heart rate, (d) affect, (e) session RPE, (f) session affect, (g) 500-m split times during the 5-km TT, (h) and reaction time as well as response accuracy for the Stroop Word-Color Test and Switching Task. RPE, heart rate, and affect were taken every 500-m during the 5-km TT. In addition, during the TT oxygen consumption (VO_2_), carbon dioxide production (VCO_2_), minute ventilation (V_E_) and respiratory exchange ratio (RER), were assessed and derived from indirect calorimetry (Fig. 1). Testing sessions were conducted within the Exercise Science Laboratory of Grove City College at the same time each day at a room temperature between 19–21 °C and a relative humidity of 35–40%.

### Participants

Thirteen recreational male distance runners participated in this study (Table 1). Participants were recruited directly from local running clubs and by community advertisement. Participants were included if they: (1) completed a 5-km run in under 25 min within the last 3 months, (2) were running a minimum of 32 km per week, (3) were between 18 and 49 years old, (4) had > 2 years of running experience, and (5) were consuming a Standard American Diet [26]. Participants were excluded from the study if they (1) had a history of smoking, (2) had any known metabolic (e.g., diabetes) or cardiovascular disease, (3) presence of orthopedic, musculoskeletal, neurological, psychiatric disorders and/or any medical conditions that prohibit exercise, (4) use of any prescription medications, and (5) following a low-carbohydrate or ketogenic diet. Participants were prohibited from using any ergogenic aids for one month preceding the study and were asked to refrain from taking any performance enhancing supplement(s) during the course of the study. Participants were instructed to refrain from caffeine and alcohol consumption for 48 h and racing or training for 24 h, and food and drink for 3 h before each exercise test. Before enrolling in the investigation, participants were fully informed of any risks and discomforts associated with the experiments prior to giving their written informed consent to participate. The experimental protocol was approved by the Institutional Review Board of Grove City College prior to implementation.

**Table 1:** Participant characteristics

Variable	Mean ± SD
Age (years)	24.8 ± 9.6
Height (cm)	178 ± 8.2
Weight (kg)	72.5 ± 8.3
BMI (kg/m^2^)	22.8 ± 1.8
Body fat (%)	10.5 ± 4.9
Fat mass (kg)	7.2 ± 4.1
FFM (kg)	64.7 ± 6.6
5-km races (total)	28.8 ± 20.5
Mean running distance/week (km)	38.1 ± 8.2
Running experience (years)	9 ± 4.6
VO_2_ Max (ml/kg/min)	60.1 ± 5.4

Participant age, anthropometric, and training status (*n* = 13). Values are mean ± SD

*BMI* body mass index, *FFM* fat-free mass, *VO*_*2*_* Max* maximum oxygen consumption

### Pretrial preparation

Participants were instructed to maintain their usual training frequency during the study intervention without increasing or decreasing the training load. The participants were instructed to maintain a training log (mode, duration, and intensity of each workout) for 1 week before the first experimental trial. They were provided with a copy of their pre-trial log and instructed to have the same training routine during the intervention period. In addition, participants were asked to record their training every week during the study (mode, duration, and intensity of each workout). Furthermore, to quantify the subject’s training session intensity, participants were asked to record their session RPE (sRPE) after every training session [26] (pre-trial and within trail), using the OMNI Walk/Run 0–10 Perceived Exertion Scale [27]. Training load for each session was calculated by sRPE x duration of session (minutes) [28]. The sum of each session’s training load provided the quantification of weekly training load. Training load was assessed each week to measure compliance.

Furthermore, participants’ habitual pre-trial and within trial dietary intake was assessed weekly using a 3-day weighed dietary record, consisting of 2 weekdays and 1 weekend day. Participants were provided with a copy of their pre-trial log and instructed to have the same dietary intake during the remainder of the study. During the familiarization session participants were given precise oral and written instructions individually on how to accurately record amounts and types of food and beverages. Participants were provided with a digital portable scale (Ozeri ZK 14-S Pronto, San Diego, CA) and instructed to weigh all food items separately if possible or to estimate the amounts. Diet information was entered into a commercial nutrient analysis software (Nutritionist Pro™, Axxya Systems, Stafford, TX).

### Familiarization and anthropometric measurements

At the first laboratory visit, all the experimental procedures were explained to the participants. The participants underwent an orientation involving practice of the 5-km TT and familiarization of the cognitive test battery, the various measurement instruments, equipment, affect measures and perceived exertion. Affect was measured using a validated 11-point Feeling Scale [29], with participants informed that their responses should reflect the affective or emotional components of the exercise and not the physical sensation of effort or strain. The OMNI Walk/Run Perceived Exertion Scale [27] was used to measure the physical perceptions of exertion for overall body (RPE-O), legs (RPE-L) and chest (RPE-C). Following the orientation session, anthropometric measures were obtained including height (cm), weight (kg), fat free mass (kg) and fat mass (% and kg). Height (cm) was measured using a physician’s scale (Detecto, Webb City, MO). Participants body mass (kg) and body composition (fat and lean mass) was measured using a Tanita bioelectrical impedance analyzer (BIA) (MC-980Uplus, Tanita Corporation of America, Arlington Heights, Illinois). Finally, the participants performed a 5-km familiarization trial on a motorized treadmill (Trackmaster TMX425C treadmill, Newton, KS).

### Maximal aerobic capacity

On the second laboratory visit, participants performed an incremental test to exhaustion on a motorized treadmill (Trackmaster). Oxygen consumption (VO_2_) and carbon dioxide production (VCO_2_) were measured using an automated metabolic analyzer system (TrueOne 2400, ParvoMedics, Sandy, UT) calibrated prior to each exercise test using standard calibration gases (16% O_2_ and 4% CO_2_). Participants wore a Polar heart rate monitor (H10, Polar Electro, Kempele, Finland) during exercise to measure heart rate. After a thorough explanation of the experimental procedures, each participant was instructed to walk on the treadmill for 3 min as a warm-up at a self-selected speed (0% grade). Immediately following the 3-min warm-up, the speed was increased to 5–8 mph for 3 min (0% grade) to achieve the participants’ comfortable running pace. After 3 min of running at 0% grade, the grade was increased 2.5% every 2 min throughout the test protocol while speed was kept constant. The treadmill test was terminated by the subject at the point of volitional exhaustion. At the end of the test, the highest average VO_2_ value recorded over a 30 s period of exercise was considered the participants VO_2max_.

### Experimental protocol

On the next three visits, participants were randomly assigned to ingest one of three beverages 60 min before the 5-km TT. This timing and dosing strategy is based on our own pilot experiments (unpublished data) showing that capillary *R-*βHB concentration peaked at 60 min after ingestion of a single bolus of the supplement. The supplement used in this study consisted of βHB-salt + Medium Chain Triglyceride (KETO//OS 2.1 Orange Dream, Pruvit, Melissa, TX, USA). Participants consumed one (KS1: 22.1 g) or two servings (KS2: 44.2 g) of the ketone supplement (βHB + MCT) powder mixed with approximately 237 ml of cold (~ 6 °C) water. One serving of the supplement contained a calculated 7 g βHB racemic salt (50% R-βHB and 50% S-βHB) and reported 7 g MCT. A complete list of the ingredients and the relative dose of each ingredient is provided in Additional file 1: Table S1. When receiving the PLA, participants consumed an equal amount of water with MiO Orange Tangerine Liquid Enhancer (0 mg caffeine, 0 kcal; Kraft Foods; MiO, Northfield, IL, USA). The ketone supplement, and PLA drink were similar in volume, texture, and appearance. The taste of the drinks was slightly different, and there remains the possibility that the participants were able to identify the drinks. In order to ensure a double-blinded design, each drink was presented to participants in an opaque sports bottle. To avoid the placebo effect in the experimental trials, we did not inform the participants about the names of the drinks and we presented all drinks as having similar ergogenic properties.

#### Blood sampling

Fingertip (capillary) blood samples for blood ketones (*R-*βHB; Precision Xtra, Abbott Diabetes Care Inc., Almeda, CA) and blood glucose (Precision Xtra, Abbott Diabetes Care Inc., Almeda, CA) concentrations were measured at baseline, 30 min post supplement ingestion (30 min; pre-cognitive test battery), 60 min post supplement ingestion (immediately before start of TT), immediately following the TT (+ 0 min), and 15 min following the TT (immediately following the cognitive test battery). Blood lactate concentration (Lactate Plus, Nova Biomedical) was assessed at baseline, 60 min post supplement ingestion, and immediately post exercise (+ 0 min). Samples were collected using a lancet following cleaning of the fingertip with an alcohol swab and then dried. The first droplet was wiped away with a cotton swab to remove any alcohol and the subsequent droplets were used for analysis.

#### 5-km running time trial

To determine exercise performance, participants performed a 5-km running TT on a motorized treadmill (TMX425C treadmill; Trackmaster, Newton, KS, USA). Before the start of the run, participants completed a 5-min self-paced warm-up run. Participants were instructed to finish the run as fast as possible. The gradient was set at 0.0% grade. Participants were provided with feedback on the distance (at regular 500-m intervals) covered during each TT and were not informed of the overall performance time until completion of the study. During the 5-km TT, participants were permitted to adjust their speed how and whenever they saw fit during the TT via control buttons located on the treadmill. The speed indicator and timing devices were concealed from the participant’s view throughout the TT. Therefore, participants regulated their treadmill pace according to their perceived exertion associated with the intensity of the exercise and their subjective feelings of their running capabilities [30]. Heart rate (Polar Electro, Kempele, Finland), RPE (RPE-Overall; RPE-Chest; RPE-Legs) and affect (Feeling Scale) were recorded at 500-m intervals during the 5KTT. Ratings of perceived exertion and affect for the entire exercise session (session RPE and session affect) were obtained 5 min following the TT. Metabolic gases were continuously collected during the entire TT using a metabolic cart for assessment of RER, VO_2_, VCO_2_, V_E_, RR, and substrate oxidation.

#### Cognitive test battery

Thirty minutes post supplement ingestion (30 min) and five minutes after each 5-km TT (+ 5 min), participants performed a battery of cognitive tests to assess executive cognitive function. A familiarization test was performed during the first laboratory visit to reduce the possibility of a learning effect. During this time researchers thoroughly explained the different cognitive tests to the participants, but data were not recorded. Executive function was measured before and after exercise using a computerized automated neuropsychological assessment metric (ANAM®) test (ANAM-4, Vista Life Sciences, USA). The ANAM® is a brief, self-directed, computerized neuropsychological assessment battery that assesses neuropsychological functioning. The ANAM software has been shown to have test–retest reliability [31]. Before each cognitive test battery, time-keeping devices such as watches and cell phones were removed, and during the task, participants did not receive any feedback on performance or time lapsed. Participants were seated in a comfortable chair in a sound-insulated room. Testing was performed under optimal conditions (i.e., appropriate lighting, as quiet as possible, and isolation from unnecessary stimuli). An identical test battery was administered before and after each trial. The battery took approximately 10 min to complete. Participants were instructed to complete the battery as quickly and accurately as possible. For each test, reaction time (in milliseconds) and reaction time for only correct responses (accuracy) were collected.

The test battery consisted of the following validated tests: the Stroop Word-Color Test (congruent and incongruent) and Switching Task (manikin and mathematical processing). The Stroop Test measures cognitive flexibility, processing speed, and executive function [32]. The cognitive mechanism involved in this task is directed to attention, and the participants must manage their attention by inhibiting one response to do something else. For the congruent Stroop Word-Color Test, a series of XXXX’s appeared on the computer screen in one of three colors (“red,’’ ‘‘blue,’’, or “green”). Participants were instructed to press the corresponding key (1 for “red”, 2 for “green” and 3 for “blue” on the keyboard based on color. For example, if the series of XXXX’s appeared in red font then participants were instructed to press 1 on the keyboard. For the incongruent Stroop Word-Color Test, a series of individual words (“RED”, “GREEN”, or “BLUE”) appeared on the computer screen in a color that did not match the name of the color depicted by the word. Participants were instructed to press the response key on the keyboard assigned to the color of the word on the screen. For example, if the word “BLUE” that was written in red ink appeared on the computer screen then participants were instructed to press 1 on the keyboard.

Next, the participants performed the Switching Task. The Switching Task was designed to measure divided attention, mental flexibility, and executive function [33]. The Switching Task requires users to alternate between two tasks: The Manikin and Mathematical Processing. Only one type problem (Manikin or Mathematical Processing) appears on the computer screen. For mathematical processing, participants were presented with a three-digit math equation (e.g., “5 + 4 − 2”) and if the sum was greater than “5” they were instructed to click the right mouse, and if the sum was less than “5” then they were instructed to press the left mouse. For the Manikin, participants were presented with an animated character (Manikin) holding a sphere in the left or right hand. If the manikin was holding the sphere in the right hand then participants were instructed to click the right mouse, and if the manikin was holding the sphere in the left hand then participants were instructed to click the left mouse. For each trial, the manikin shifts positions, so that it may be facing towards the viewer, away from the viewer, or to the side. Cognitive test battery was comprised of *n* = 10 participants for the Stroop Word-Color Test (congruent and incongruent) and *n* = 11 participants for the Switching Task (manikin and mathematical processing) due to technical issues in *n* = 3 and *n* = 2, respectively.

#### Statistical analysis

Statistical analyses were performed using SPSS version 24.0 (SPSS Inc., Chicago, IL). Statistical significance was set a priori at *p* < 0.05. Descriptive statistics were calculated for all variables. Normality and absence outliers were verified by using the Shapiro–Wilk test, normality plots, and residual plots. Performance, physiological, and perceptual data collected during the 5-km TT (5-km running time, mean exercise heart rate, RER, VO_2_, VCO_2_, V_E_, RR, carbohydrate and fat oxidation rates, affect, RPE-Chest, RPE-Legs, RPE-Overall, session RPE and session affect) were analyzed using a one-way repeated measures analysis of variance (ANOVA). A 3 (condition, KS1 vs KS2 vs PLA) × 10 (every 500 m) repeated measures ANOVA was conducted to assess the effect of time, treatment, and interaction between time and treatment, on heart rate, affect, RPE-Chest, RPE-Legs, RPE-Overall, and time covered at each 500-m interval during the 5-km TT. A 3 (condition, KS1 vs KS2 vs PLA) × 5 (rest, 30 min post ingestion, 60 min post ingestion, immediately post exercise, and 15 min post-TT) repeated measures ANOVA was conducted to assess the effect of time, treatment, and interaction between time and treatment, on capillary glucose and ketones. A 3 (condition, KS1 vs KS2 vs PLA) × 3 (baseline, 60 min post supplement ingestion, and immediately post exercise) repeated measures ANOVA was conducted to assess the effect of time, treatment, and interaction between time and treatment, on capillary lactate. A 3 (condition, KS1 vs KS2 vs PLA) × 2 (time, pre vs post) repeated measures ANOVA was conducted to assess the effect of time, treatment, and interaction between time and treatment, on the Stroop Word-Color Test and Switching Task. A one-way repeated measures ANOVA was used to analyze differences over time for training load and nutrient intake before and during the intervention. The smallest worthwhile change (SWC) was predetermined to be 0.65%. This is midway between the smallest worthwhile change in day-to-day variability in competitive middle distance and distance runners [34] and the estimated coefficient of variation in our laboratory [25, 30, 35]. Post hoc analyses of significant main and interaction effects were conducted where appropriate using the Bonferonni adjustment to determine which conditions were significantly different. The assumption of sphericity was confirmed using Mauchly's test. Greenhouse–Geisser epsilon corrections were used when the sphericity assumption was violated. Partial-eta squared (η^2^p) was used to report effect size with 0.01 considered small, 0.06 medium, and 0.14 large effects. All data are reported as Mean ± SD.

## Results

### Dietary and exercise adherence

Participants’ pre-trial dietary intake averaged 3068 ± 764 Kcals and comprised 349 ± 123 g (44.7%) of carbohydrate, 123 ± 33 g (16.4%) of protein, and 125 ± 33 g (38.1%) of fat, consistent with the Standard American Diet (Additional file 1: Table S2) [24]. No significant changes in dietary characteristics were observed during the course of the study. Furthermore, participants’ pre-trial weekly training load averaged 1448 ± 1002 au. with no significant differences (*p* = 0.433) throughout.

### Blood βHB, glucose, and lactate

**Fig. 2:**
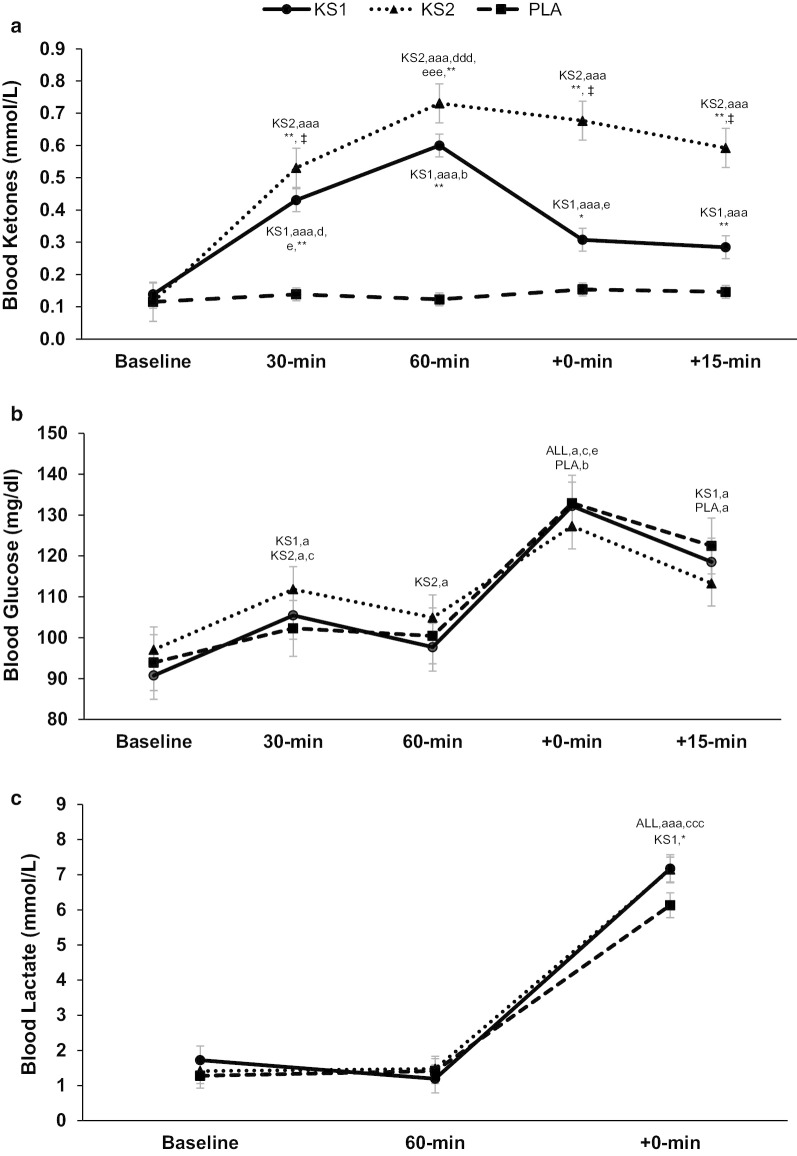
Blood metabolites. Impact of exogenous ketone supplementation on blood **a** R-β-hydroxybutyrate, **b** glucose, and **c** lactate (*n* = 13). Values are mean ± SE. One Dose Beta Hydroxybutyrate Salt and Medium Chain Triglycerides, KS1; Double Dose Beta Hydroxybutyrate Salt and Medium Chain Triglycerides, KS2; Flavored Matched Control, PLA; All Groups, ALL; *, significantly different from PLA (*p* < 0.05); **, significantly different from PLA (*p* < 0.001); ‡, significantly different between KS groups (*p* < 0.05); a, significantly different from baseline (*p* < 0.05); aaa, Significantly different from baseline (*p* < 0.0001); b, significantly different from 30-min (*p* < 0.05); c, significantly different from 60-min (*p* < 0.05); ccc, significantly different from 60-min (*p* < 0.0001); d, significantly different from + 0 min (*p* < 0.05); ddd, significantly different from + 0-min (*p* < 0.0001); e, significantly different from + 15-min (*p* < 0.05); eee, significantly different from + 15-min (*p* < 0.0001)

Blood *R*-βHB had a significant time X treatment interaction (*p* < 0.001). Follow up tests indicated that blood *R*-βHB for the KS2 condition was significantly higher 30 min post supplementation compared to KS1 and PLA (*p* = 0.031 and < 0.001, respectively). Furthermore, blood *R*-βHB was significantly higher (*p* < 0.001) 30 min after beverage ingestion in KS1 compared to PLA. Additionally, blood *R*-βHB 60 min post supplementation was higher for KS1 and KS2 compared to the PLA (all *p*’s < 0.001). Finally, blood *R*-βHB for the KS2 condition was higher both immediately post exercise (+ 0 min) (*p* = 0.002 and < 0.001, respectively) and post cognitive test (+ 15 min) (*p* = 0.002 and < 0.001) compared to KS1 and PLA. In addition, blood *R*-βHB for the KS1 condition was higher both immediately post exercise (+ 0 min) (*p* < 0.001) and post cognitive test (+ 15 min) (*p* < 0.001) compared to PLA (Additional file 1: Table S3; Fig. 2a).

Blood glucose in the KS1 and KS2 treatments increased significantly from baseline to 30 min post drink (*p* = 0.006 and 0.009, respectively), decreased significantly from 30 to 60 min post drink only in the KS 2 treatment (*p* = 0.049), increased significantly from 60 min to immediately post exercise (+ 0 min) in all three treatments (*p* = 0.013, 0.017, 0.038, respectively for KS1, KS2, and PLA), and decreased significantly from immediately post exercise (+ 0 min) to post cognitive test (+ 15 min) in all three treatments (*p* = 0.013, 0.040, 0.011; respectively for KS1, KS2, and PLA; Fig. 2b; Additional file 1: Table S3). However, there was no significant effect for treatment (*p* = 0.830) or time X treatment interaction (*p* = 0.355).

Blood lactate increased significantly from baseline to immediately post exercise in all conditions (+ 0 min; *p* = 0.001; Fig. 2c; Additional file 1: Table S3). There was a significant time X treatment interaction (*p* = 0.020). Follow up tests indicated that blood lactate was significantly higher in KS1 (*p* = 0.024) and non-significantly, but trending higher in KS2 (*p* = 0.056) immediately post exercise (+ 0 min) compared to PLA (Fig. 2c).

#### Physiological, perception, and physical performance response

**Fig. 3:**
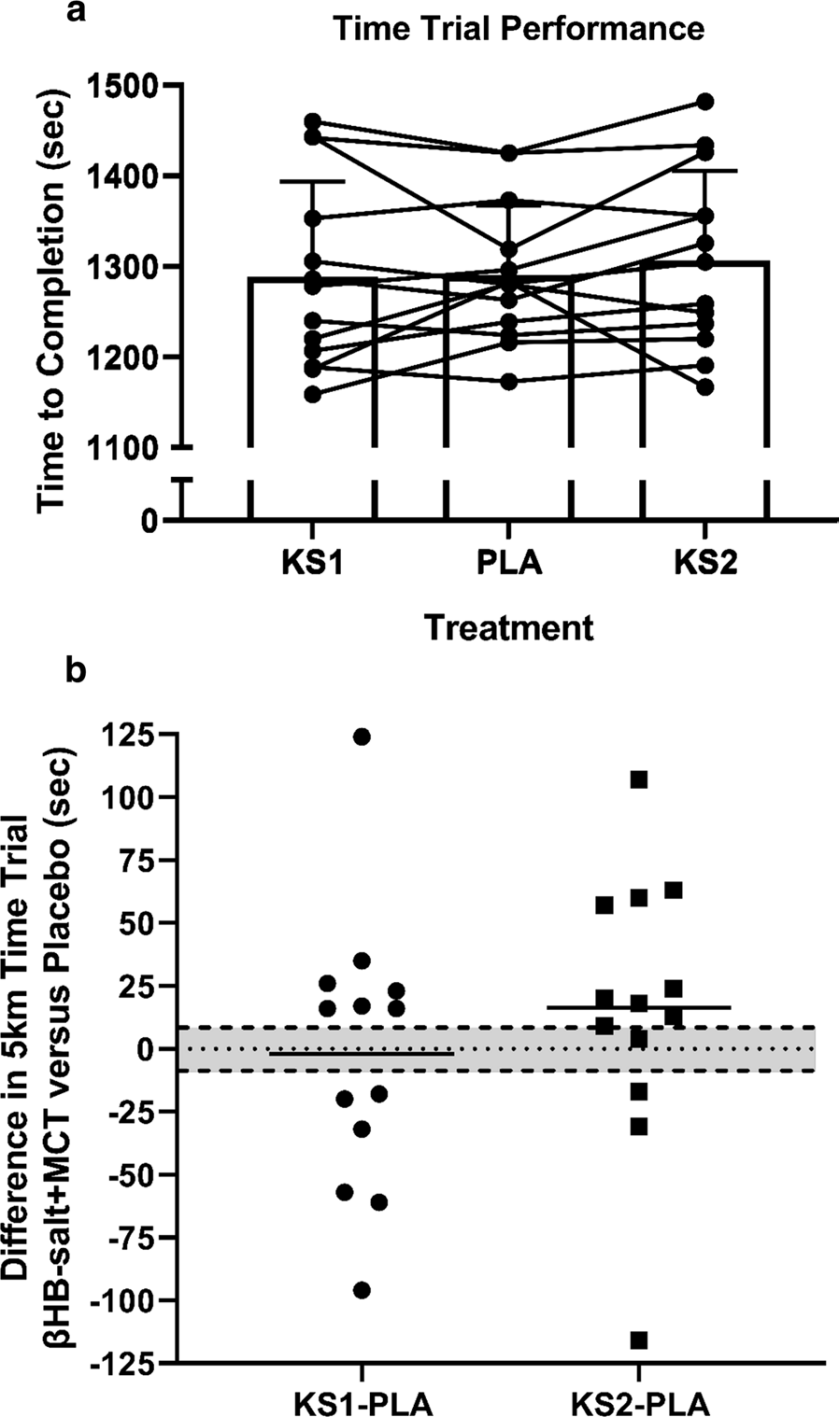
5-km time trial performance. **a** Individual and average 5-km performance time responses to exogenous ketone supplements and placebo ingestion (*n* = 13). **b** Difference in 5 k time trial between ketone supplements and placebo ingestion with smallest worthwhile difference (SWD) range shaded in grey. Values are mean ± SD. One Dose Beta Hydroxybutyrate Salts and Medium Chain Triglycerides, KS1; Two Doses Beta Hydroxybutyrate Salts and Medium Chain Triglycerides, KS2; Flavored Matched Control, PLA

No trial order effect was observed for the 5-km TT performance between visit 3, 4, and 5 (*p* = 0.342). The ketone supplement had no effect on 5-km run TT performance (KS1, 1289.0 ± 104.9 s; KS2, 1307.3 ± 98.8 s; PLA, 1291.1 ± 77.1 s; *p* = 0.386; η^2^p = 0.076; Fig. 3; Table 2). Overall, the KS1 and KS2 conditions resulted in non-significant ~ 2 s (0.16%) faster and ~ 16 s (1.2%) slower TT performance compared to PLA, respectively. However, six subjects ran faster and seven slower in KS1, while three subjects ran faster and eight subjects slower than PLA which were greater than SWG. There was no difference in VO_2_, VCO_2_, RER, V_E_, RR, estimated carbohydrate oxidation rate, estimated fat oxidation rate, heart rate, affect, RPE-C, RPE-L, and RPE-O between KS1, KS2, and PLA during the 5-km TT (Table 2). Running speeds for each 500-m split during the 5-km TT did not differ between trials but did increase progressively throughout the TT (main effect for time, *p* < 0.001).

**Table 2:** Metabolic, respiratory, heart rate, perceptual, and physical performance response.

Variable	KS1	KS2	PLA	*p *value	η^2^p
Mean VO_2_ (L/min)	3.5 ± 0.6	3.5 ± 0.6	3.5 ± 0.6	0.706	0.029
Mean VCO_2_ (L/min)	3.4 ± 0.7	3.3 ± 0.6	3.3 ± 0.6	0.336	0.087
RER	0.97 ± 0.02	0.96 ± 0.03	0.95 ± 0.04	0.172	0.136
Mean VO_2_ (ml/kg/min)	49.0 ± 6.3	48.2 ± 4.7	48.4 ± 5.6	0.571	0.046
V_E_ (L/min)	106.2 ± 21.4	102.8 ± 19.3	99.8 ± 28.8	0.570	0.046
RR (bpm)	42.7 ± 6.5	42.1 ± 6.9	43.7 ± 6.7	0.155	0.144
*Mean carbohydrate oxidation (g/min)*	4.2 ± 0.9	4.0 ± 0.8	3.9 ± 0.8	0.191	0.129
*Mean fat oxidation (g/min)*	0.20 ± 0.12	0.25 ± 0.15	0.29 ± 0.23	0.316	0.092
Heart rate (b/min)	176.0 ± 8.3	176.8 ± 7.9	175.2 ± 9.3	0.575	0.045
Affect	0.6 ± 2.1	0.4 ± 2.1	0.7 ± 2.2	0.587	0.043
RPE-C	4.8 ± 1.9	4.9 ± 2.2	5.0 ± 2.0	0.524	0.052
RPE-L	5.4 ± 2.0	5.0 ± 2.3	5.4 ± 1.9	0.315	0.092
RPE-O	5.2 ± 1.9	5.0 ± 2.1	5.3 ± 2.0	0.390	0.075
Session RPE	6.2 ± 1.7	6.5 ± 1.8	6.8 ± 2.1	0.141	0.151
Session affect	0.5 ± 2.3	− 0.8 ± 2.9	− 0.1 ± 2.7	0.088	0.183
Time (s)	1289.0 ± 104.9	1307.3 ± 98.8	1291.1 ± 77.1	0.386	0.076

Gas exchange, respiratory rate (RR), calculated oxidation rates, heart rate (HR), affect, rate of perceived exertion (RPE), and time to completion data during 5-km time trail (5-km TT; *n* = 13). Values are Mean ± SD. One Dose Beta Hydroxybutyrate Salt and Medium Chain Triglycerides, KS1; Double Dose Beta Hydroxybutyrate Salt and Medium Chain Triglycerides, KS2; Flavored Matched Control, PLA; oxygen consumption, VO_2_; carbon dioxide production, VCO_2_; heart rate, HR; respiratory exchange ratio, RER; ventilation, V_E_; respiratory rate, RR; rate of perceived exertion, RPE; rate of perceived exertion for overall body, RPE-O; rate of perceived exertion for chest, RPE-C; rate of perceived exertion for leg RPE-L. *Italicized:* Did not correct for ketone oxidation (Frayn et al. 1980s; Cox et al.)

#### Cognitive performance

**Fig. 4:**
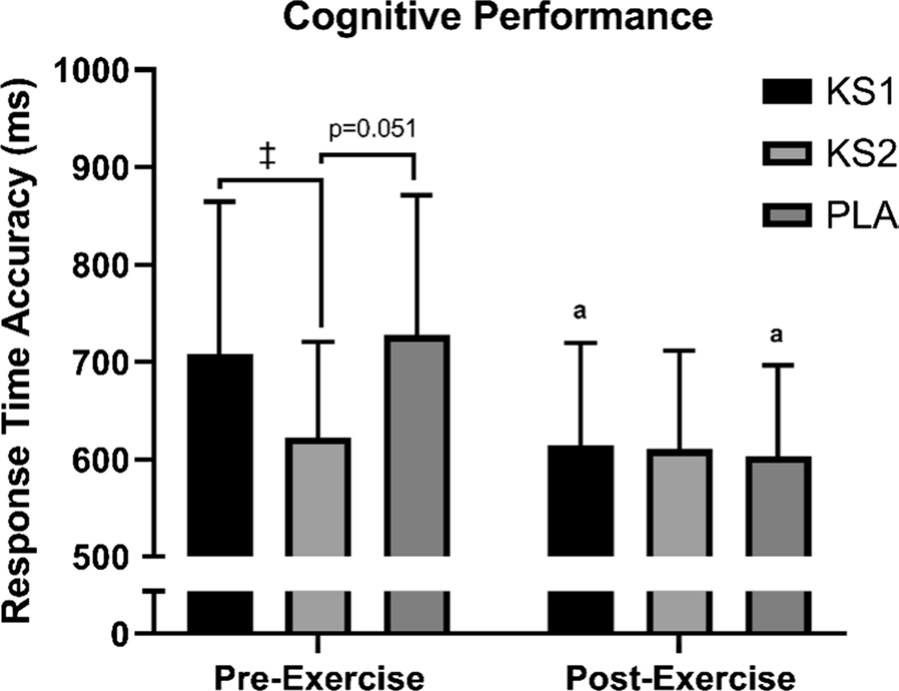
Reaction time accuracy. Reaction time accuracy was assessed via Stroop incongruent assessment (*n* = 13). Values are mean ± SD. One Dose Beta Hydroxybutyrate Salt and Medium Chain Triglycerides, KS1; Double Dose Beta Hydroxybutyrate Salt and Medium Chain Triglycerides, KS2; Flavored Matched Control, PLA. ‡, significantly different between KS groups (*p* < 0.05); a, significantly different from Pre-TT (*p* < 0.05)

Average response accuracy and reaction time for congruent and incongruent Stroop trials at each testing time for the KS1, KS2, and PLA condition are presented in Fig. 4 and Additional file 1: Table S4. No significant main effects of treatment or treatment X Time interaction were found for reaction time or response accuracy for congruent trials (*p* > 0.05; Additional file 1: Table S4). No significant main effects for treatment or treatment X time interaction were found for response time for the incongruent trial (*p* = 0.266 and 0.077, respectively). However, a significant time X treatment interaction was found for average response accuracy in the Stroop incongruent test (*p* = 0.043). Post-hoc analyses revealed that the KS1 and PLA conditions showed a significant faster reaction time (*p* = 0.002 and 0.010, respectively) from pre-TT (30 min) to post-TT (+ 5 min) while the KS2 treatment indicated and a non-significant faster reaction time (*p* = 0.582). Additionally, response time accuracy was significantly faster in KS2 pre-TT (30 min) compared to KS1 (*p* = 0.018), with none significant trends for faster response in KS2 compared to PLA pre-TT (*p* = 0.051). A significant main effect of time for response accuracy and reaction time during congruent and incongruent trials was observed (*p* = 0.001), with improved executive function from pre (30 min) to post test (+ 5 min) (*p* = 0.001) in all conditions. Performance time (reaction time and response accuracy) on the Switching task, decreased significantly from pre-TT (30 min) to post-TT (+ 5 min; *p* = 0.039 and 0.026, respectively), but there was no significant effect for treatment (*p* = 0.120 and 0.116, respectively) or treatment X time interaction (*p* = 0.154 and 0.254, respectively; Additional file 1: Table S4).

## Discussion

A consistent finding across all exogenous ketone bodies is their ability to induce rapid and widespread changes in metabolism, including consistent changes in circulating metabolic concentrations, ketones, glucose, lactate, amino acids, and/or free-fatty acids. Unsurprisingly, all analyses to date have demonstrated that exogenous ketones significantly elevate circulating blood ketones, although to differing extents pending the exogenous agent [5, 8, 13–23]. Synthetic 1,3 BD and/or esters raise *R*-βHB to a greater extent than ketone salts [3, 6]. However, few studies have looked at the dosing effect of exogenous ketones [6, 8]. Here we demonstrate that two dosages of βHB + MCT result in greater *R-*βHB peak concentrations (60mins post ingestion: KS2: 0.73 mM, KS1 0.60 mM) and significant impact on duration of *R-*βHB elevations, illustrating a clear dosing effect on circulating ketone pharmacokinetics with βHB + MCT administration. Importantly, many exogenous ketone formulations come as racemic mixtures (50% *R*-βHB: 50% *S*-βHB) due to lower production cost of utilizing racemic precursors [1]. However, all commercial ketone meters and wet-lab assays only detect *R-*βHB and most don’t test for acetoacetate, limiting detecting of total ketone load in ours and other ketone evaluations. None the less, *R*- and *S-*βHB have differential metabolic consequences with the latter being less readily oxidizable [6, 36], suggesting that determining *R*-βHB are likely more relevant in assessing metabolic substrate induce energetic shifts on performance [37]. Ketone elevations are often accompanied by significant or directional reductions in basal blood glucose or post-exercise glucose elevations, with rare exceptions [22]. While we did not observe significant administration effect on blood glucose in the present analyses, we did see non-significant blunting of post-exercise glucose elevations with KS2 (31.1% rise, compared to 45% in KS1 and 40.5% rise in PLA), suggesting a potential dosing effect of βHB + MCT on blood glucose. However, more inconsistent are the ability of exogenous ketones to blunt exercise lactate production. Ketone salts and/or βHB + MCT have not shown an ability to attenuate exercise lactate productions, consistent with the present findings [13–16, 25]. However, attenuated lactate production is more consistently observed with ester administration [8, 18, 20], although it does not always reach significance [21, 22]. We found that neither administration nor dose of βHB + MCT influenced heart rate, RR, RER, VO_2_, VCO_2_, VE, or estimated metabolic oxidation rates. HR has elevated with ketone salts administration [15, 16] or reductions in HR with ester administration [18, 20], with the vast majority consistent with our findings across exogenous ketone supplements [19, 21–23, 25]. While most exogenous ketone evaluations have not observed changes in RER in agreement with our observations, some analyses have observed differences [8, 13, 14]. These differences in RER have been extrapolated into shifts in fat and carbohydrate oxidations [8, 13], as were calculated in the present analyses. However, limiting all these oxidation calculation are observations of RER of βHB and AcAc are 0.89 and 1.00, respectively, directly confounding calculations of glucose and fat oxidation [38], but potentially explaining the shifts in RER observed in some evaluations [8, 13, 14]. Attempts to control for exogenous ketone administration in oxidation calculations should be approached with caution as it can often be difficult at present and lead to over-estimations in calculated fat-oxidation [8].

Exogenous ketones bodies have been explored in a various exercise performance trials including MCTs [5], ketone salts [13–16], esters [8, 17–21], and 1,3BD [22, 23]. Across seventeen separate MCT exercise performance analyses, only two and five analyses demonstrated ergogenic and ergolytic effect, respectively, attributed primarily to gastrointestinal distress [5]. Four analyses (3/4 on performance) have been conducted with ketone salts with either neutral [14] or negative [13] impact on endurance performance, and neutral impact on sprint performance [16]. Lack of efficacy across ketone salts trials have been largely attributed to an inability to reach sufficient circulating *R-*βHB concentrations hypothesized to be important for facilitating sufficient energetic needs and metabolic shifts [7, 39]. Eleven separate analyses have been conducted on *R/S* 1,3 Butanediol (1,3-BD, [22, 23]), Beta-hydroxybutyrate *R* 1,3 Butanediol (Monoester; [8, 17–19, 21]), and/or *R/S* 1,3 Butanediol Acetoacetate (Diester; [20]) with three [8, 17, 21], six [18, 19, 21–23], two [20, 21] showing positive, neutral, or negative impacts on exercise performance, respectively. These positive findings across 1,3 BD and Monoester were attributed to sufficiently elevated *R*-βHB (≥ 2 mM *R-*βHB), reduced lactate production, and attenuated glycogen utilization [8, 17, 21]. However, neutral and/or negative effects maybe explained in part due to gastrointestinal symptoms [18, 19, 21, 37], while other hypothesis include insufficient circulating *R-*βHB [19–23], choice of exercise, administration without carbohydrates [23], and/or placebo comparison. Our group is the only to have conducted analyses on βHB + MCT which demonstrated non-significant impact on run performance [25]. Here we repeated those findings, while also demonstrating that dosing did not affect run performance potentially explained by insufficient ability to reach hypothesize ergogenic substrate threshold [39] as gastrointestinal symptoms were not indicated in this study cohort (unreported). However, consistent with prior findings [19, 25], we also found divergent effect of ketone administration run performance across subjects which were greater than the SWD, suggesting responders and non-responders.

Ketone-induced perceptual and cognitive outcomes have become a growing area of interest due to ketones multifaceted impact on the brain. We report that βHB + MCT administration nor dose impact perceptual outcomes in line with independent observations with other ketogenic agents [14, 15, 18–20, 22, 23, 25], but discrepant from others [16, 21]. Only two separate analyses have evaluated cognition performance showing positive impact with monoester [18] and neutral impact of ketone salts [13]. We demonstrate faster response time accuracy in KS2 prior to exercise. Interestingly, post exercise, all groups had equivalent cognitive function to pre-exercise KS2 group, suggesting a dosing response on basal cognitive function equivalent to the exercise-induced effect on PLA group. However, limiting our interpretation of these findings are a lack of pre-supplement cognitive function evaluations across groups.

## Conclusion

βHB + MCT novel ketone formulation demonstrated a dosing effect on blood *R*-βHB and cognitive function, an administration response on blood lactate, and no administration or dosing impact across physiologic, perceptual, and physical performance parameters. However, clear responders and non-responders were found. To our knowledge, this is the first analyses comparing the multi-component dose–response of βHB + MCT across blood metabolites, gaseous exchange, respiratory rate, heart rate, perception, and physical and cognitive performance parameters. Further analyses should consider similar dose–response impact across other exogenous ketones administration, as well as divergent factors influencing responders versus non-responders, to help understand how to optimally administer these agents across health, disease, and performance applications.

## Supplementary information

Additional file 1:
** Table S1.** Ketone supplement ingredients; **Table S2.** Nutrient intake; **Table S3.** Blood metabolites; **Table S4.** Cognitive Function Scores.

## Data Availability

The datasets used and/or analyzed during the current study are available from the corresponding author on reasonable request.

## Abbreviations

TT: Time trial
KS1: One dose beta hydroxybutyrate salt and medium chain triglycerides
KS2: Double dose beta hydroxybutyrate salt and medium chain triglycerides
PLA: Placebo
VO_2_: Oxygen consumption
VCO_2_: Carbon dioxide production
HR: Heart rate
RER: Respiratory exchange ratio
VE: Ventilation
RR: Respiratory rate
RPE-O: Rating of perceived exertion for overall body
RPE-C: Rating of perceived exertion for chest
RPE-L: Rating of perceived exertion for legs
sRPE: Session rating of perceived exertion
MCT: Medium-chain triglycerides
βHB: Beta hydroxybutyrate
ANAM: Automated neuropsychological assessment metric
BMI: Body mass index
FFM: Fat-free mass
VO_2max_: Maximum oxygen consumption

## References

[1] Poff AM, Koutnik AP, Egan B (2020). Nutritional ketosis with ketogenic diets or exogenous ketones: features, convergence, and divergence. Curr Sports Med Rep.

[2] Cahill GF (2006). Fuel metabolism in starvation. Annu Rev Nutr.

[3] Kesl SL, Poff AM, Ward NP, Fiorelli TN, Ari C, Van Putten AJ, Sherwood JW, Arnold P, D'Agostino DP (2016). Effects of exogenous ketone supplementation on blood ketone, glucose, triglyceride, and lipoprotein levels in Sprague–Dawley rats. Nutr Metab (Lond).

[4] Koutnik AP, D'Agostino DP, Egan B (2019). Anticatabolic effects of ketone bodies in skeletal muscle. Trends Endocrinol Metab.

[5] Clegg ME (2010). Medium-chain triglycerides are advantageous in promoting weight loss although not beneficial to exercise performance. Int J Food Sci Nutr.

[6] Stubbs BJ, Cox PJ, Evans RD, Santer P, Miller JJ, Faull OK, Magor-Elliott S, Hiyama S, Stirling M, Clarke K (2017). On the metabolism of exogenous ketones in humans. Front Physiol.

[7] Evans M, Cogan KE, Egan B (2017). Metabolism of ketone bodies during exercise and training: physiological basis for exogenous supplementation. J Physiol.

[8] Cox PJ, Kirk T, Ashmore T, Willerton K, Evans R, Smith A, Murray AJ, Stubbs B, West J, McLure SW (2016). Nutritional ketosis alters fuel preference and thereby endurance performance in athletes. Cell Metab.

[9] Newman JC, Verdin E (2017). Beta-hydroxybutyrate: a signaling metabolite. Annu Rev Nutr.

[10] Koutnik AP, Poff AM, Ward NP, DeBlasi JM, Soliven MA, Romero MA, Roberson PA, Fox CD, Roberts MD, D'Agostino DP (2020). Ketone bodies attenuate wasting in models of atrophy. J Cachexia Sarcopenia Muscle.

[11] Cotter DG, Schugar RC, Crawford PA (2013). Ketone body metabolism and cardiovascular disease. Am J Physiol Heart Circ Physiol.

[12] Poff AM, Koutnik AP, Egan KM, Sahebjam S, D'Agostino D, Kumar NB (2017). Targeting the Warburg effect for cancer treatment: Ketogenic diets for management of glioma. Semin Cancer Biol.

[13] O'Malley T, Myette-Cote E, Durrer C, Little JP (2017). Nutritional ketone salts increase fat oxidation but impair high-intensity exercise performance in healthy adult males. Appl Physiol Nutr Metab.

[14] Rodger S, Plews D, Laursen P, Driller M (2017). Oral B-hydroxybutyrate salt fails to improve 4-minute cycling performance following submaximal exercise. J Sci Cycl.

[15] Evans M, Patchett E, Nally R, Kearns R, Larney M, Egan B (2018). Effect of acute ingestion of beta-hydroxybutyrate salts on the response to graded exercise in trained cyclists. Eur J Sport Sci.

[16] Waldman HS, Basham SA, Price FG, Smith JW, Chander H, Knight AC, Krings BM, McAllister MJ (2018). Exogenous ketone salts do not improve cognitive responses after a high-intensity exercise protocol in healthy college-aged males. Appl Physiol Nutr Metab.

[17] Cox PJ. The effects of a novel substrate on exercise energetics in elite athletes, dissertation. Oxford University; 2013.

[18] Evans M, Egan B (2018). Intermittent running and cognitive performance after ketone ester ingestion. Med Sci Sports Exerc.

[19] Evans M, McSwiney FT, Brady AJ, Egan B (2019). No benefit of ingestion of a ketone monoester supplement on 10-km running performance. Med Sci Sports Exerc.

[20] Leckey JJ, Ross ML, Quod M, Hawley JA, Burke LM (2017). Ketone diester ingestion impairs time-trial performance in professional cyclists. Front Physiol.

[21] Smith A. The power of substrate: an examination of the physiological basis for and functional impact of a novel nutritional intervention for sports performance, dissertation. University of Bath, Department of Health; 2017.

[22] Scott BE, Laursen PB, James LJ, Boxer B, Chandler Z, Lam E, Gascoyne T, Messenger J, Mears SA (2019). The effect of 1,3-butanediol and carbohydrate supplementation on running performance. J Sci Med Sport.

[23] Shaw DM, Merien F, Braakhuis A, Plews D, Laursen P, Dulson DK (2019). The effect of 1,3-butanediol on cycling time-trial performance. Int J Sport Nutr Exerc Metab.

[24] Shan Z, Rehm CD, Rogers G, Ruan M, Wang DD, Hu FB, Mozaffarian D, Zhang FF, Bhupathiraju SN (2019). Trends in dietary carbohydrate, protein, and fat intake and diet quality among US adults, 1999–2016. JAMA.

[25] Prins PJ, Koutnik AP, D'Agostino DP, Rogers CQ, Seibert JF, Breckenridge JA, Jackson DS, Ryan EJ, Buxton JD, Ault DL (2020). Effects of an exogenous ketone supplement on five-kilometer running performance. J Hum Kinet.

[26] Lovell TW, Sirotic AC, Impellizzeri FM, Coutts AJ (2013). Factors affecting perception of effort (session rating of perceived exertion) during rugby league training. Int J Sports Physiol Perform.

[27] Robertson RJ, Goss FL, Dube J, Rutkowski J, Dupain M, Brennan C, Andreacci J (2004). Validation of the adult OMNI scale of perceived exertion for cycle ergometer exercise. Med Sci Sports Exerc.

[28] Foster C, Florhaug JA, Franklin J, Gottschall L, Hrovatin LA, Parker S, Doleshal P, Dodge C (2001). A new approach to monitoring exercise training. J Strength Cond Res.

[29] Hardy CJR, Rejeski W (1989). Not what, but how one feels: the measurement of affect during exercise. J Sport Exerc Psychol.

[30] Prins PJ, Goss FL, Nagle EF, Beals K, Robertson RJ, Lovalekar MT, Welton GL (2016). Energy drinks improve five-kilometer running performance in recreational endurance runners. J Strength Cond Res.

[31] Vincent AS, Roebuck-Spencer TM, Fuenzalida E, Gilliland K (2018). Test–retest reliability and practice effects for the ANAM General Neuropsychological Screening battery. Clin Neuropsychol.

[32] Djamshidian A, O'Sullivan SS, Lees A, Averbeck BB (2011). Stroop test performance in impulsive and non impulsive patients with Parkinson's disease. Parkinsonism Relat Disord.

[33] Shattuck NL, Shattuck LG, Matsangas P: Combat effectiveness and sleep patterns in US Marines. In: Human factors and ergonomics society annual meeting Los Angeles, CA. SAGE Publications; 2016. p. 886–90.

[34] Schubert MM, Astorino TA, Azevedo JL (2013). The effects of caffeinated "energy shots" on time trial performance. Nutrients.

[35] Prins PJ, Welton GL, Ryan EJ, Majchrowicz C, Althausen J, Fijal J, Sorek N, Dallatore T, Ault DL (2018). Effects of energy drink functional ingredients on running performance. J Exerc Nutr.

[36] Desrochers S, David F, Garneau M, Jette M, Brunengraber H (1992). Metabolism of R- and S-1,3-butanediol in perfused livers from meal-fed and starved rats. Biochem J.

[37] Stubbs BJ, Koutnik AP, Poff AM, Ford KM, D'Agostino DP (2018). Commentary: ketone diester ingestion impairs time-trial performance in professional cyclists. Front Physiol.

[38] Frayn KN (1983). Calculation of substrate oxidation rates in vivo from gaseous exchange. J Appl Physiol Respir Environ Exerc Physiol.

[39] Egan B, D'Agostino DP (2016). Fueling performance: ketones enter the mix. Cell Metab.

