# Supplementary material for: Dose response of a novel exogenous ketone supplement on physiological, perceptual and performance parameters
Source: Nutr Metab (Lond). 2020 Sep 29;17:81. doi: 10.1186/s12986-020-00497-1 (PMC7523040; doi:10.1186/s12986-020-00497-1)
Supplement: Supplementary file 1 — Additional file 1: Table S1. Ketone supplement ingredients; Table S2. Nutrient intake; Table S3. Blood metabolites; Table S4. Cognitive Function Scores. [file 12986_2020_497_MOESM1_ESM.docx]

**Supplemental Digital Content (SDC)**

**Supplementary Tables and Figures**

TABLE S1. Ketone Supplement Ingredients

|  | KS1 (22.1 g) | KS2 (44.2 g) |
| --- | --- | --- |
| Total Calories (Kcal) | 110 | 220 |
| *R*-Beta Hydroxybutyrate (*R*-βHB) (g) | *3.5* | *7* |
| *S*-Beta Hydroxybutyrate (*S-*βHB) (g) | *3.5* | *7* |
| Medium-Chain Triglycerides (MCT; g) | 7 | 14 |
| Sodium (g) | 1.42 | 2.84 |
| Potassium (mg) | 50 | 100 |
| Total Carbohydrates (g) | 3 | 6 |
| Sugars (g) | 2 | 4 |
| Protein (g) | 1 | 2 |
| Calcium (mg) | 440 | 880 |

Beta hydroxybutyrate salts and medium chain triglycerides supplement compositions. Data are reported values based on product labeling and calculations. Calories, Kcal; R-Beta Hydroxybutyrate, R-βHB; S-Beta Hydroxybutyrate, S-βHB; Medium-Chain Triglycerides, MCT; KS1, βHB-salts and MCTs 1x dose; KS2, βHB-salts and MCTs 2x dose. *Italicized* = estimated grams of Beta Hydroxybutyrate (βHB) from 1) calculation of the molecular equivalence of the mineral load to (βHB), 2) calculation of the difference between total and product weight and non-βHB weight, and 3) 50:50 racemic *R-*βHB:*S-*βHB composition.

TABLE S2. Nutrient intake

| Diet | Pre-Trial | Week 1 | Week 2 | Week 3 | P-Value |
| --- | --- | --- | --- | --- | --- |
| Calories (Kcals) | 3068.1 ± 764.7 | 3049.0 ± 876.0 | 3161.7 ± 1057.1 | 2693.2 ± 888.2 | 0.252 |
| Carbohydrate (g) | 349.6 ± 123.4 | 371.9 ± 138.2 | 376.5 ± 162.1 | 310.6 ± 137.9 | 0.177 |
| Protein (g) | 123.5 ± 33.7 | 119.1 ± 47.3 | 123.9 ± 37.4 | 110.5 ± 42.7 | 0.544 |
| Fat (g) | 125.9 ± 35.1 | 118.4 ± 28.3 | 122.8 ± 50.8 | 108.2 ± 36.2 | 0.583 |
| Carbohydrate (%) | 44.7 ± 7.2 | 48.1 ± 6.5 | 47.3 ± 8.4 | 45.9 ± 7.2 | 0.430 |
| Protein (%) | 16.4 ± 2.5 | 15.9 ± 3.9 | 16.7 ± 3.7 | 16.7 ± 2.5 | 0.886 |
| Fat (%) | 38.1 ± 7.4 | 36.1 ± 5.0 | 36.0 ± 6.9 | 37.4 ± 7.5 | 0.636 |
| Cholesterol (mg) | 429.7 ± 258.2 | 355.5 ± 169.2 | 391.4 ± 232.8 | 389.9 ± 199.8 | 0.431 |
| Saturated fat (g) | 42.6 ± 14.4 | 36.8 ± 11.1 | 40.0 ± 17.0 | 35.4 ± 11.8 | 0.483 |
| Monounsaturated fat (g) | 32.8 ± 10.4 | 30.8 ± 8.6 | 32.2 ± 16.5 | 27.3 ± 13.6 | 0.613 |
| Polyunsaturated fat (g) | 18.5 ± 6.4 | 19.4 ± 7.7 | 18.8 ± 8.9 | 17.5 ± 12.3 | 0.910 |
| Sugar (g) | 119.3 ± 59.2 | 137.2 ± 88.3 | 128.9 ± 72.7 | 119.8 ± 66.0 | 0.686 |

Participants adhered to a Standard American Diet [26] nutrient intake through the intervention timeline (*n* = 13). Values are Mean ± SD.

TABLE S3. Blood Metabolites

|  | Time | | | | | Differences from baseline | | | | *P*-Value |
| --- | --- | --- | --- | --- | --- | --- | --- | --- | --- | --- |
|  | **Baseline** | **30 min** | **60 min** | **+0 min** | **+15 min** | **30 min** | **60 min** | **+0 min** | **+15 min** |  |
| Blood *R*-β-hydroxybutyrate (mmol/L) |  |  |  |  |  |  |  |  |  |  |
| KS1 | 0.14 ± 0.07 | 0.43 ± 0.2^aaa, d, e, **^ | 0.60 ± 0.2^aaa, ddd, eee, **^ | 0.31 ± 0.1^aaa, **^ | 0.28 ± 0.1^aaa, **^ | 0.29 ± 0.14 | 0.46 ± 0.14 | 0.17 ± 0.08 | 0.15 ± 0.09 | Time, *P* <0.001  Condition, *P* <0.001  Interaction, *P* <0.001 |
| KS2 | 0.12 ± 0.04 | 0.53 ± 0.2^aaa, **, ‡^ | 0.73 ± 0.2^aaa, b, **^ | 0.68 ± 0.3^aaa, e, **, ‡^ | 0.59 ± 0.3^aaa, **,^ ^‡^ | 0.41 ± 0.17 | 0.61 ± 0.21 | 0.56 ± 0.32 | 0.48 ± 0.31 |  |
| PLA | 0.12 ± 0.04 | 0.14 ± 0.05 | 0.12 ± 0.04 | 0.15 ± 0.07 | 0.15 ± 0.07 | 0.02 ± 0.06 | 0.01 ± 0.03 | 0.04 ± 0.08 | 0.03 ± 0.06 |  |
| Blood Glucose (mg/dl) |  |  |  |  |  |  |  |  |  |  |
| KS1 | 90.8 ± 8.6 | 105.5 ± 15.0^a^ | 97.7 ± 10.9 | 132.2 ± 38.1^a, c, e^ | 118.5 ± 32.5^a^ | 14.7 ±15.9 | 6.9 ± 13.5 | 41.5 ± 40.5 | 27.8 ± 33.4 | Time, *P* <0.001  Condition, *P* = 0.830  Interaction, *P* = 0.355 |
| KS2 | 97.1 ± 13.0 | 111.9 ± 19.3^a, c^ | 104.9 ± 13.0^a^ | 127.3 ± 27.1^a, c, e^ | 113.3 ± 27.9 | 14.8 ± 17.0 | 7.8 ± 9.2 | 30.2 ± 29.9 | 16.2 ± 26.9 |  |
| PLA | 93.9 ± 10.7 | 102.3 ± 14.1 | 100.5 ± 18.7 | 132.9 ± 41.9^a, b, c, e^ | 122.5 ± 37.7^a^ | 8.4 ± 15.4 | 6.5 ± 16.7 | 39.0 ± 43.9 | 28.5 ± 38.9 |  |
| Blood Lactate (mmol/L) |  |  |  |  |  |  |  |  |  |  |
| KS1 | 1.7 ± 1.3 | - | 1.2 ± 0.5 | 7.2 ± 2.5^aaa, ccc, *^ | - | - | -0.53 ± 1.3 | - | 5.5 ± 2.3 | Time, *P* <0.001  Condition, *P* = 0.223  Interaction, *P* = 0.020 |
| KS2 | 1.4 ± 0.9 | - | 1.5 ± 0.5 | 7.1 ± 2.5^aaa, ccc^ | - | - | 0.06 ± 0.9 | - | 5.7 ± 2.1 |  |
| PLA | 1.3 ± 0.5 | - | 1.4 ± 1.1 | 6.1 ± 2.2^aaa, ccc^ | - | - | 0.14 ± 0.9 | - | 4.8 ± 2.1 |  |

*C*apillary blood glucose, *R*-β-hydroxybutyrate, and lactate were assessed across five timepoints (*n*=13). Values are Mean **±** SD. One Dose Beta Hydroxybutyrate Salt and Medium Chain Triglycerides, KS1; Double Dose Beta Hydroxybutyrate Salt and Medium Chain Triglycerides, KS2; Flavored Matched Control, PLA. *, significantly different from PLA (p<0.05); **, significantly different from PLA (p<0.001); ‡, significantly different between KS groups (p<0.05); a, significantly different from baseline (p<0.05); aaa, significantly different from baseline (p<0.0001); b, significantly different from 30-min (p<0.05); c, significantly different from 60-min (p<0.05); ccc, significantly different from 60-min (p<0.0001); d, significantly different from +0min (p<0.05); ddd, significantly different from +0-min (p<0.0001); e, significantly different from +15-min (p<0.05); eee, significantly different from +15-min (p<0.0001).

TABLE S4. Cognitive Function Scores.

| **Test Variable** | **KS1** | | | **KS2** | | | **PLA** | | | ***P*-Value** |
| --- | --- | --- | --- | --- | --- | --- | --- | --- | --- | --- |
|  | **Pre-TT**  **30-min** | **Post-TT**  **+5-min** | **Post-Pre** | **Pre-TT**  **30-min** | **Post-TT**  **+5-min** | **Post-Pre** | **Pre-TT**  **30-min** | **Post-TT**  **+5-min** | **Post-Pre** |  |
| **Stroop, Congruent (ms) (N = 10)** | | | | | | | | | | |
| **Mean Reaction Time** | 589.6 ± 79.0 | 536.2 ± 83.2^aa^ | -53.4 ± 34.1 | 554.7 ± 86.7 | 527.4 ± 104.1^a^ | 27.3 ± 34.6 | 567.5 ± 82.1 | 530.5 ± 73.1^a^ | 37.0 ± 39.9 | Time, *P*=0.001  Condition, *P*=0.565  Interaction, *P*=0.274 |
| **Mean Reaction Time Correct** | 586.1 ± 77.4 | 530.7 ± 86.6^aa^ | 55.4 ± 38.3 | 557.8 ± 87.2 | 523.8 ± 107.6^a^ | 33.9 ± 37.9 | 569.8 ± 83.1 | 524.7 ± 72.4^a^ | 45.1 ± 44.5 | Time, *P*=0.001  Condition, *P*=0.686  Interaction, *P*=0.426 |
| **Stroop, Incongruent (ms) (N = 10)** | | | | | | | | | | |
| **Mean Reaction Time** | 708.8 ± 159.9 | 622.6 ± 100.8^a^ | 86.1 ± 75.5 | 626.8 ± 95.8 | 607.0 ± 106.3 | 19.8 ± 66.2 | 730.1 ± 148.2 | 604.6 ± 92.1^a^ | 125.5 ± 129.9 | Time, *P*=0.001  Condition, *P*=0.266  Interaction, *P*=0.077 |
| **Mean Reaction Time Correct** | 707.9 ± 156.7 | 615.0 ± 104.4^a^ | 92.9 ± 69.3 | 622.6 ± 98.8^‡^ | 610.5 ± 101.3 | 12.0 ± 66.5 | 728.3 ± 143.3 | 603.3 ± 93.3^b^ | 125.0 ± 123.5 | Time, *P*=0.001  Condition, *P*=0.261  Interaction, *P*=0.043 |
| **Switching, Manikin and Mathematical Processing Test (ms) (N = 11)** | | | | | | | | | | |
| **Mean Reaction Time** | 1916.3 ± 610 | 1577.4 ± 366.7^a^ | 338.9 ± 481.5 | 1699.6 ± 398.6 | 1563.9 ± 499.7 | 135.7 ± 308.6 | 1585.0 ± 345.2 | 1463.3 ± 444.2 | 121.7 ± 251.1 | Time, *P*=0.039  Condition, *P*=0.120  Interaction, *P*=0.154 |
| **Mean Reaction Time Correct** | 1902.3 ± 613.4 | 1572.6 ± 368.7^a^ | 329.7 ± 489.5 | 1695.2 ± 399.2 | 1548.4 ± 495.0 | 146.7 ± 315.5 | 1586.6 ± 330.2 | 1437.8 ± 391.9^a^ | 148.8 ± 185.9 | Time, *P*=0.026  Condition, *P*=0.116  Interaction, *P*=0.254 |

Mean reaction time and number of correct responses were evaluated via Stroop Congruent, Stroop Incongruent, Switching, Manikin and Mathematical Processing Tests (*n*=10-11). Values are Mean ± SD. One Dose Beta Hydroxybutyrate Salt and Medium Chain Triglycerides, KS1; Double Dose Beta Hydroxybutyrate Salt and Medium Chain Triglycerides, KS2; Flavored Matched Control, PLA; TT, Time Trial; a, significantly different from Pre-TT (30-min) (*p*<0.05); aa, significantly different from Pre-TT (30-min) (*p* <0.001); ‡, significantly different between KS groups (p<0.05)
